# Supplementary material for: The causal role of circulating inflammatory markers in osteoporosis: a bidirectional Mendelian randomized study
Source: Front Immunol. 2024 Jul 18;15:1412298. doi: 10.3389/fimmu.2024.1412298 (PMC11291241; doi:10.3389/fimmu.2024.1412298)
Supplement: Supplementary file 1 [file DataSheet_1.zip › supplementary material/OSTEOPOROSIS-CIM/GCST90274813.tsv.gz_forest.pdf]

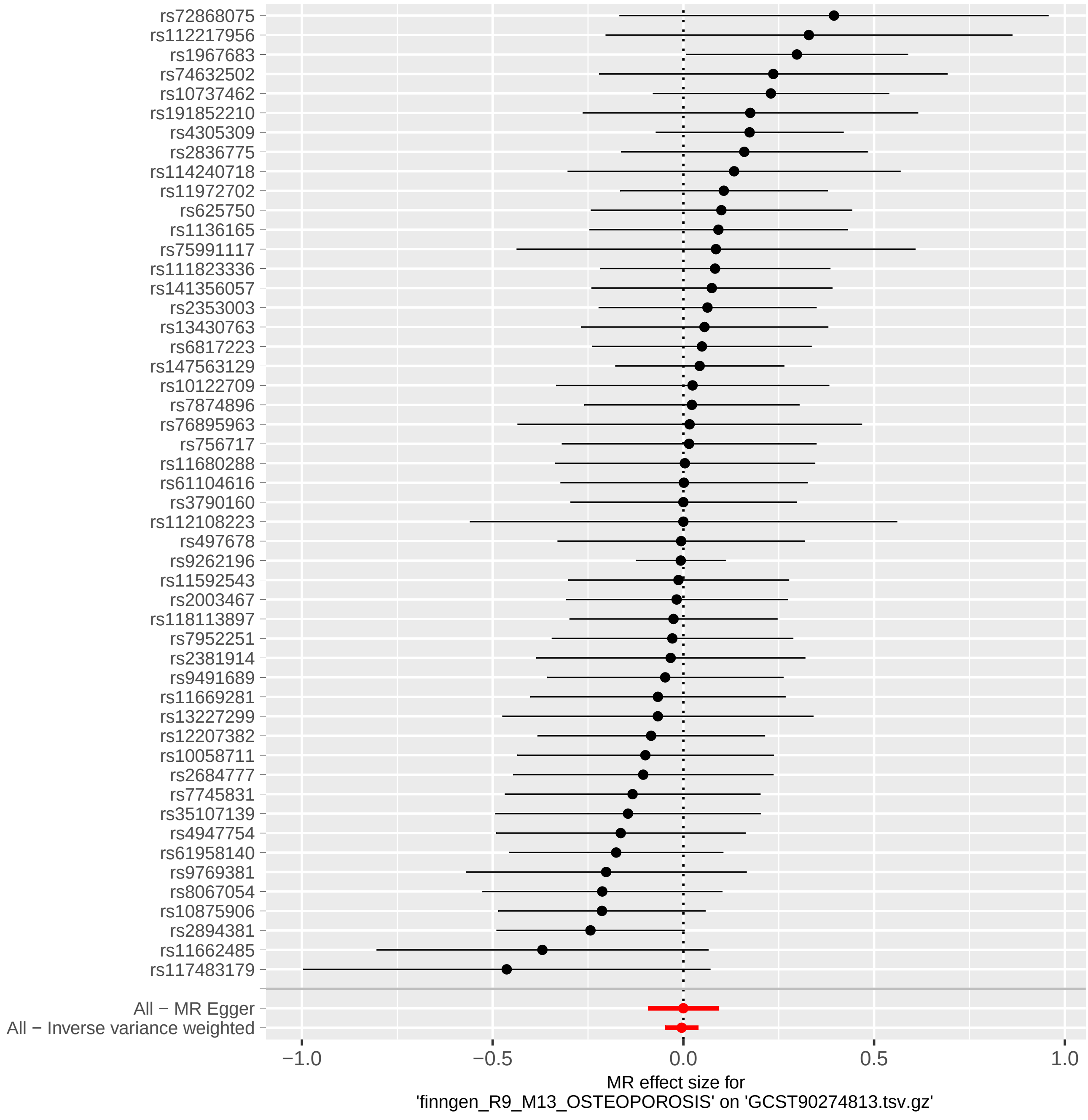
rs72868075
rs112217956
rs1967683
rs74632502
rs10737462
rs191852210
rs4305309
rs2836775
rs114240718
rs11972702
rs625750
rs1136165
rs75991117
rs111823336
rs141356057
rs2353003
rs13430763
rs6817223
rs147563129
rs10122709
rs7874896
rs76895963
rs756717
rs11680288
rs61104616
rs3790160
rs112108223
rs497678
rs9262196
rs11592543
rs2003467
rs118113897
rs7952251
rs2381914
rs9491689
rs11669281
rs13227299
rs12207382
rs10058711
rs2684777
rs7745831
rs35107139
rs4947754
rs61958140
rs9769381
rs8067054
rs10875906
rs2894381
rs11662485
rs117483179
All − MR Egger
All − Inverse variance weighted
−1.0
−0.5
0.0
0.5
1.0
MR effect size for
'finngen_R9_M13_OSTEOPOROSIS' on 'GCST90274813.tsv.gz'
